# The usefulness of wide excision assisted by a computer navigation system and reconstruction using a frozen bone autograft for malignant acetabular bone tumors: a report of two cases

**DOI:** 10.1186/s12885-018-4971-8

**Published:** 2018-10-24

**Authors:** Kensaku Abe, Norio Yamamoto, Katsuhiro Hayashi, Akihiko Takeuchi, Shinji Miwa, Kentaro Igarashi, Hiroyuki Inatani, Yu Aoki, Takashi Higuchi, Yuta Taniguchi, Hirotaka Yonezawa, Yoshihiro Araki, Hiroyuki Tsuchiya

**Affiliations:** Department of Orthopaedic Surgery, Graduate School of Medical Sciences, Kanazawa University, 13-1 Takara-machi, Kanazawa, 920-8641 Japan

**Keywords:** Ewing sarcoma, Dedifferentiated chondrosarcoma, Acetabulum, Computer navigation system, Frozen autograft, Reconstruction

## Abstract

**Background:**

Difficult resection of tumors from regions with complex local anatomy, such as the pelvis and sacrum, is likely to result in inadequate surgical margins (intralesional or marginal); this is because three-dimensional osteotomy is difficult particularly around the acetabulum. Additionally, removal of the joint makes reconstruction very difficult; thus, retention of good function also becomes difficult. In musculoskeletal oncology, computer navigation systems are still not widely used to prevent tumor-positive margins. We performed wide excision with guidance from a computer navigation system and reconstruction using frozen bone autografts for malignant pelvic bone tumors in two patients, and we obtained excellent functional and oncological outcomes. Here we present these patients and discuss our approach.

**Case presentation:**

Case 1: A 12-year-old girl presented with Ewing sarcoma of the left pelvis (PI-II). We performed wide excision assisted by a computer navigation system with the osteotomy of the load surface of acetabulum and reconstruction using a frozen bone autograft. At the final follow-up, she showed excellent function and was alive without the disease. Moreover, she did not have osteoarthritis of the left hip joint.

Case 2: A 71-year-old woman presented with dedifferentiated chondrosarcoma of the right pelvis (PII-III). We performed wide excision assisted by a computer navigation system with osteotomy avoiding load surface of the acetabulum and reconstruction using a frozen bone autograft; there was no tumor at the load surface. At the final follow-up, she showed good function, was alive without the disease, and did not have osteoarthritis of the left hip joint.

**Conclusions:**

Wide excision assisted by a computer navigation system and reconstruction using a frozen bone autograft are very useful for the management/treatment of extremely difficult cases such as malignant pelvic bone tumors, particularly those including the acetabulum.

## Background

Difficult resection of tumors from regions with complex local anatomy, such as the pelvis and sacrum, or from regions that require complex multiplanar or geometric bony resection, is likely to result in inadequate surgical margins (intralesional or marginal) [1]. This can drastically affect a patient’s outcome, and it may result in local tumor recurrence in up to 92% of cases [2–8]. The use of computer navigation systems in spinal surgery, arthroplasty, deformity correction, and trauma has improved surgical precision by providing more detailed intraoperative information and guidance [9–14]. However, computer navigation systems are yet to be widely used in musculoskeletal oncology, although many authors have also reported encouraging results, such as a good intraoperative orientation, precise surgical margins, low rates of local recurrence and complications, and good functional outcomes because of the possibility of pelvic reconstruction [15–19].

**Fig. 1:**
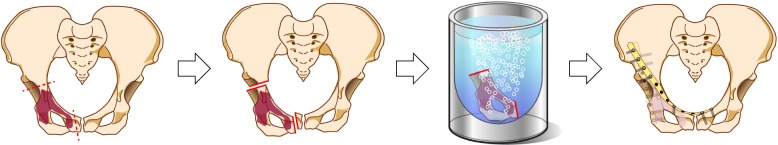
Pelvic osteotomy and free-freezing method. The tumor is first excised by osteotomy. Then, after peeling off the soft tissue, we performed curettage of the tumor and drilling to prevent bone breakage. After freezing in liquid nitrogen, osteosynthesis was performed using plates, screws, and a bone graft

Pelvic resection called PII according to Enneking and Dunham [20] and periacetabular resection present a unique surgical challenge as no specific reconstruction approach has been shown to be superior [21]. Although many reconstructive options exist, the best reconstructive option for these patients is still being debated. The options include endoprosthetic reconstruction [22–24], hip transposition [25, 26], iliofemoral arthrodesis [27, 28], biological reconstruction (using allografts or autografts from the tibia, fibula, iliac crest, or pelvis) [29, 30], and hip rotationplasty [31]. In our institution, we have developed a novel surgical procedure involving reconstruction using frozen autografts [32, 33] (Fig. 1). This method has been shown to be associated with particularly excellent functional and oncological outcomes [32–38].

We performed wide excision with guidance from a computer navigation system and reconstruction using frozen bone autografts for malignant pelvic bone tumors in two patients, and we obtained excellent functional and oncological outcomes. Here, we present these patients and discuss our approach.

### Operative methods using computer navigation

We used OrthoMap software (Stryker, Kalamazoo, Michigan) as a computer navigation system. In the preoperative plan, the plot was based on computed tomography (CT) (case 1) or both CT and gadolinium-enhanced magnetic resonance imaging (MRI) (case 2). The CT and MRI slice thicknesses were 1.5 and 4 mm, respectively. We used only CT for case 1 because chemotherapy was extremely effective and the soft tissue mass disappeared; we used both CT and MRI for case 2 because chemotherapy was less effective and the soft tissue mass remained. We confirmed that the tumor did not grow in size 1 week before surgery, and then, we used the image acquired before chemotherapy. If the tumor grows, then, it is considered that the image should be the latest one. This process took 2–3 h. During the operation, the navigation station was located on the opposite side of the operator to eliminate any obstacles between the camera and the patient’s/instrument’s tracker. The patient’s tracker was placed on the iliac crest, and registration points were registered until the error was < 1 mm using the anterior superior iliac spine, sacroiliac joint, and exposed iliac crest as the registration points with comparisons with 3D imaging. Once we determined the osteotomy site, bones were cut using a chisel linked with navigation, a bone saw, and a threadwire saw (T-saw; Depuy AcroMed, Inc., Cleveland, OH) [39–42]. Although determining navigation settings requires 30–60 min, doubts about determining osteotomy sites were dramatically reduced. The resection margin was pathologically confirmed using a small sample collected from a preserved host tissue.

### Operative methods using frozen autografts

The surgical procedure has previously been described [32]. To kill the tumor cells, rapid freezing and slow thawing were performed. In particular, the tumor-bearing bone was frozen in liquid nitrogen for 20 min, thawed at room temperature, and distilled in water for 15 min. When the tumor-bearing bone was frozen, moisture was removed, the bone marrow cavity was curettaged, and the bone was punctured in multiple places using a Kirschner wire to prevent fracture. The frozen autograft was replaced with the reconstruction. Bone graft or cement was used for mechanical support when necessary.

## Case presentations

### Case 1

**Fig. 2:**
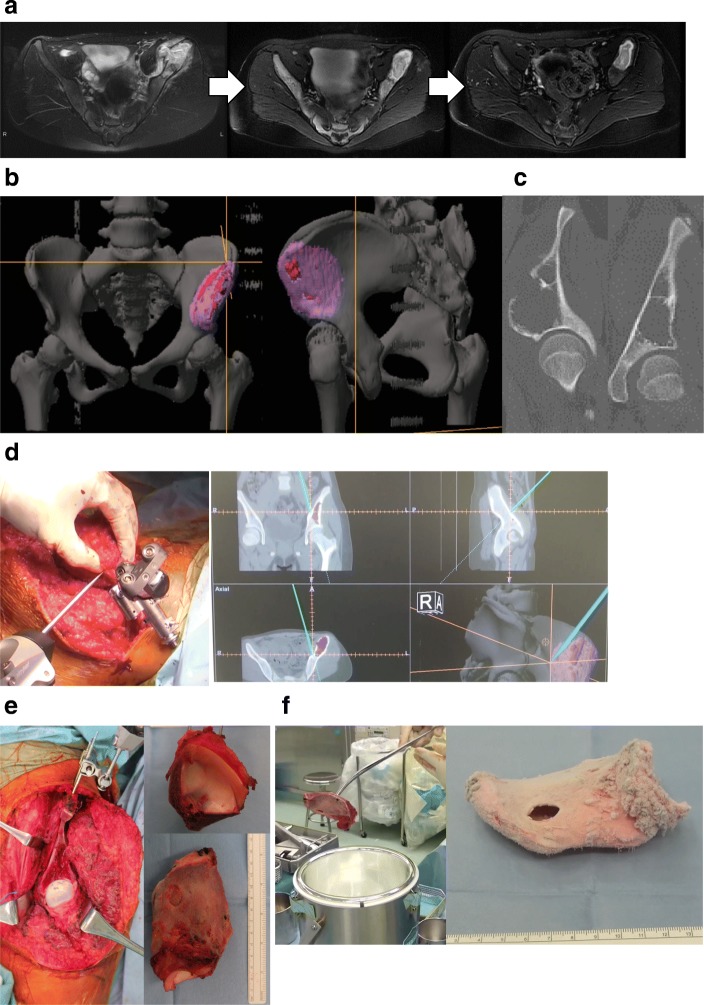
Preoperative assessments in case 1: A 12-year-old girl with Ewing sarcoma of the pelvis (PI-II). **a** Preoperative magnetic resonance imaging. **b**) Preoperative plan involving the use of a computer navigation system. **c**) Preoperative computed tomography. **d** Checking the margin using a computer navigation system. **e** After osteotomy. **f** Freezing in liquid nitrogen

**Fig. 3:**
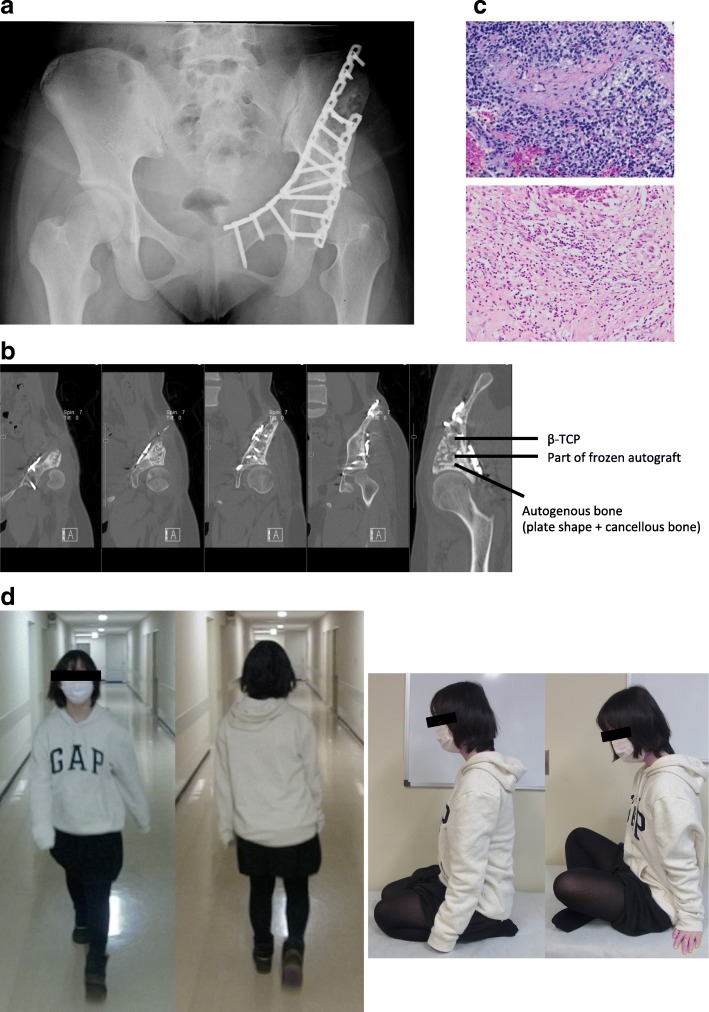
Postoperative assessments in case 1: A 12-year-old girl with Ewing sarcoma of the pelvis (PI-II). **a** Radiograph obtained at 23 months after reconstruction. **b** Computed tomography at 23 months after reconstruction. Bone union is observed, and osteoarthritis is not yet observed. **c** Pathological findings at biopsy (upper panel) and post-neoadjuvant chemotherapy (lower panel). After neoadjuvant chemotherapy, a necrotic change was observed. **d** She can walk almost normally, and there are no limitations in the sitting posture

A 12-year-old girl presented with Ewing sarcoma of the left pelvis (PI-II). Neoadjuvant chemotherapy (4 courses of ifosfamide + etoposide and 4 courses of vincristine + doxorubicine + cyclophosphamide) was administered, and the extra-skeletal mass disappeared on MRI. Wide excision and reconstruction were performed. We planned osteotomy including the load surface of acetabulum assisted by a computer navigation system and reconstruction using a frozen bone autograft. The patient’s tracker was placed on the iliac crest; the error after registration was within 1 mm. Subsequently, by pulling the lower limbs, a gap of approximately 2 cm was made in the joint space and osteotomy was performed without dislocation of the hip joint (Fig. 2). In this case, navigation was particularly helpful in osteotomy of the acetabulum. Although osteotomy under direct view is possible via dislocation of the hip joint, it involves the risk of femoral head necrosis, and this cannot be confirmed from the cartilage surface. In addition, X-ray image alone cannot provide the orientation. Reconstruction was performed using plates and an artificial bone graft and autograft (normal iliac bone) to fill the cavity for the defect of the load surface of acetabulum after tumor curettage. The resection margins were free of tumor, and this was pathologically confirmed in a small sample collected from preserved host tissue. Pathological evaluation of curettage of cancellous bone indicated almost total necrosis of the tumor, which was classified as grade III/IV according to the Rosen and Huvos evaluation system [43]. After the completion of postoperative chemotherapy (three courses of ifosfamide + etoposide, 1 course of vincristine + doxorubicine + cyclophosphamide, and two courses of vincristine + cyclophosphamide), the patient was free of the disease. Her X-ray did not show osteoarthritis of the hip joint; she could walk normally with some claudication, and there were no limitations in the sitting posture (no limitation of the range of motion of the hip and knee joint) at her 23-month follow-up (Fig. 3). Moreover, we evaluated the outcome at the 23-month follow-up using the Musculoskeletal Tumor Society (MSTS) score [44], Toronto Extremity Salvage Score (TESS) [45], and 36-item Short-form Health Survey (SF-36) [46, 47]. Her MSTS score was 86.7 and TESS was 86.5. Additionally, her physical component summary, mental component summary, and role-social component summary scores in the SF-36 were 39.4, 59.9, and 44.1, respectively (each summary had a mean of 50 and a standard deviation of 10, according to data from 2007 for healthy Japanese individuals [47]).

### Case 2

**Fig. 4:**
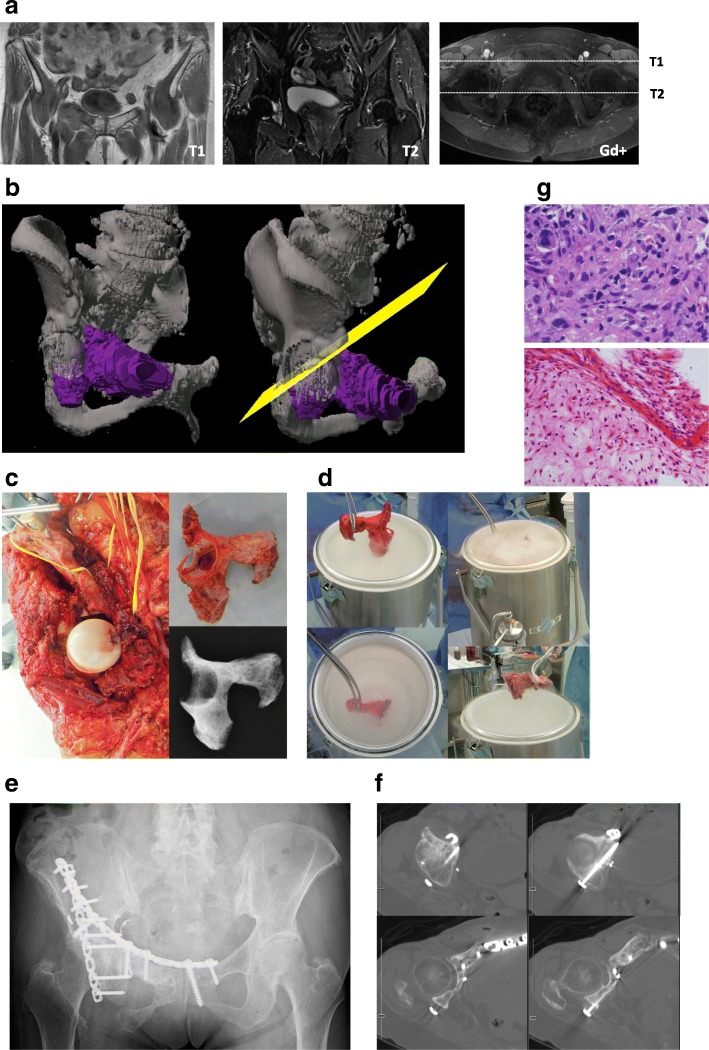
Preoperative and postoperative assessments in case 2: A 71-year-old woman with dedifferentiated chondrosarcoma of the pelvis (PII-III). **a**) Preoperative magnetic resonance imaging. **b** Preoperative plan involving the use of a computer navigation system. The osteotomy line was planned as indicated by the yellow surface. **c** After osteotomy **d** Procedure of freezing in liquid nitrogen. **e** Radiograph obtained at 33 months after reconstruction. **f** Computed tomography at 33 months after reconstruction. Bone union is observed, and osteoarthritis is not very prominent. **g** Pathological findings at biopsy (upper panel) and post-neoadjuvant chemotherapy (lower panel). After neoadjuvant chemotherapy, a necrotic change was observed

A 71-year-old woman presented with dedifferentiated chondrosarcoma of the right pelvis (PII-III). The initial pathological diagnosis was osteosarcoma, and thus, neoadjuvant chemotherapy (3 courses of cisplatin + doxorubicine) was administered. The chemotherapy caused marked shrinkage and ossification of the tumor. Wide excision and reconstruction were performed. We planned osteotomy avoiding load surface of the acetabulum, in which there was no tumor, assisted by a computer navigation system. The patient’s tracker was placed on the iliac crest, and the error after registration was within 1 mm. In this case, navigation was also particularly useful for osteotomy of the acetabulum. The location and orientation of osteotomy were extremely important for preserving the load surface, but similar to other methods, it is currently impossible to precisely achieve this. Reconstruction was performed using plates and a frozen bone autograft (Fig. 4). The resection margins were free of tumor, and this was pathologically confirmed in a small sample collected from preserved host tissue. Pathological evaluation of curettage of the tumor, which was classified as grade II/IV according to the Rosen and Huvos evaluation system. The patient did not undergo postoperative chemotherapy as the final diagnosis was dedifferentiated chondrosarcoma (resistant to chemotherapy), and her physical status was limited. However, she was free of the disease her X-ray did not show osteoarthritis of the hip joint. She could walk almost normally with a cane at her 33-month follow-up. At that follow-up, her MSTS score was 63.3 and TESS was 68.8. Additionally, her physical component summary, mental component summary, and role-social component summary scores in the SF-36 were 26.0, 58.8, and 33.1, respectively.

## Discussion

Consistent with the findings of previous studies [15–19], both our cases successfully underwent excision with tumor-free margins associated with the use of a computer navigation system. Previous reports by Jeys L et al. [19], Cho HS et al. [48], and Wong KC et al. [49] included 31, 10, and 12 patients, respectively, and these patients underwent resection under computer navigation for a pelvic or sacral bone tumor. These reports mentioned that computer navigation was a safe technique with no specific complications. In these reports, the rates of tumor-free margins were 90.3% (28/31), 100% (10/10), and 100% (12/12), respectively. However, the local recurrence rates were 9.7% (3/31), 20% (2/10), and 25% (3/12), respectively. In the first report, the bone resection margin was clear, but the soft-tissue resection margin was intralesional in all cases. Although computer navigation allows more accurate resection in patients with pelvic and sacral tumors, a clear resection margin alone does not appear to prevent local recurrence. The use of navigation can only improve the accuracy of bony resection and avoid inadvertent perforation of the tumor with osteotomy. The narrow soft-tissue margins associated with the pelvis cannot be improved by using navigation [19]. Our patients had tumor-free margins for not only bone but also soft tissue, and they are presently free of the disease. However, these results were not completely accurate as one of the disadvantages of reconstruction using a frozen autograft is the inability to perform histological analysis of the whole specimen. This is one of the limitations of our approach. In contrast to traditional intraoperative 2D X-ray image guidance, the technique completely utilizes CT and MR images and 3D models for real-time osteotomy guidance. This may improve surgical accuracy by decreasing intralesional resection and maximally preserving normal bone tissue. In these cases, partial acetabular preservation was performed. A perfect fit cannot be achieved, except by recycling the autograft, and this is the only method for reconstruction after the osteotomy adhered, as observed in this case.

Periacetabular reconstruction is challenging and no single reconstructive strategy has been proven to be superior [50]. Saddle prosthesis was initially popular because of its ease of insertion and its proposed benefits of preservation of length and hip mobility as compared with fusion; however, with reports of poor function and high complication rates, the technique has fallen out of favor [51]. Later generations of endoprosthesis that relied upon fixation to the remnant ilium have also suffered from high rates of instability, loosening, and infection [52–55]. Anatomical reconstruction of a functional hip joint seems desirable [50]. In our cases, using a frozen autograft, we were able to realize the anatomical reconstruction, and good results were obtained. Conversely, several complications were reported, such as infection, fracture, non-union, limb length discrepancy, and bone absorption [34–37]. However, no complications were noted at the 23- and 33-month short-term follow-up visits. Although freezing, in our cases, extended to the joints, not all surfaces of the joints were frozen. If the joint capsule and ligaments are solid, then stability is good, and the possibility of long-term maintenance exists.

The postoperative assessment scores (MSTS score, TESS, and SF-36) in our cases and in cases from other reports are summarized in Table 1. When our cases were compared with previous cases, our younger case (case 1) showed superior outcomes, while our older case (case 2) showed outcomes that were neither superior nor inferior. Moreover, the mental component summary score in the SF-36 was high in both our cases, suggesting that the patients were satisfied with the operation. Problems such as the degree of improvement in function with age and the transition to osteoarthritis in the future can vary, but in order to obtain better function and satisfaction, reconstruction using a frozen bone autograft is suggested to be very useful.

**Table 1:** Postoperative assessment scores (MSTS score, TESS, and SF-36) on literature review

Study	Surgical procedures	Number of patients	MSTS score (%)	TESS (%)	SF-36		
					PCS	MCS	RCS
Fujiwara T, et al. [56]	Endoprosthesis	11	42	N/A	N/A	N/A	N/A
	Hip transposition	4	49	N/A	N/A	N/A	N/A
	Iliofemoral arthrodesis	2	68	N/A	N/A	N/A	N/A
	Frozen autograft	1	50	N/A	N/A	N/A	N/A
Fuchs B, et al. [28]	Iliofemoral arthrodesis	21	71	76	N/A	N/A	N/A
	Hip transposition	11	25	52	N/A	N/A	N/A
Kim HS, et al. [57]	Pasteurized autograft	11	61	N/A	N/A	N/A	N/A
Cottias P, et al. [58]	Saddle prosthesis	17	57	58	N/A	N/A	N/A
Abdu A, et al. [22]	Custom-made hemipelvic endoprosthesis	35	70	N/A	N/A	N/A	N/A
Jaiswal PK, et al. [23]	Custom-made hemipelvic endoprosthesis	98	N/A	60	N/A	N/A	N/A
*Our case: 12-year-old girl*	Frozen autograft	1	87	87	39	60	44
*Our case: 71-year-old woman*	Frozen autograft	1	63	69	26	59	33

*MSTS* Musculoskeletal Tumor Society; *TESS* Toronto Extremity Salvage Score; *SF-36* 36-item Short-form Health Survey; *PCS* Physical component summary; *MCS* Mental component summary; *RCS* Role-social component summary; *N/A* Not available

In conclusion, our patients with malignant acetabular bone tumors were successfully treated with our approach. They were free of the disease and had excellent function. Wide excision with guidance from a computer navigation system and reconstruction using a frozen bone autograft appear to be very useful for the treatment of a malignant acetabular bone tumor.

## Abbreviations

CT: computed tomography
MRI: magnetic resonance imaging
MSTS: Musculoskeletal Tumor Society
SF-36: 36-item Short-form Health Survey
TESS: Toronto Extremity Salvage Score
